# Longitudinal Associations Between Exposure to Physical Interparental Violence and Dating Violence in Young Adulthood and the Moderating Role of Sex, Socioeconomic Status, and Antisociality

**DOI:** 10.1177/08862605251318290

**Published:** 2025-02-26

**Authors:** Andrés E. Montiel, Margot Peeters, Gonneke W. J. M. Stevens

**Affiliations:** 1 Department of Interdisciplinary Social Science, Faculty of Social and Behavioural Sciences, Utrecht University, The Netherlands

**Keywords:** dating violence, young adulthood, family violence, social learning

## Abstract

Dating violence (DV) is a widespread problem that undermines the well-being of young adults. Consistent with social learning theory, exposure to interparental violence (IV) and childhood maltreatment have been identified as risk factors for DV perpetration and victimization. However, former research on these associations is mainly U.S.-based, cross-sectional, and focused on physical DV. To address these gaps in the literature, the aims of this study were twofold: first, to assess whether exposure to physical IV during childhood was associated with physical and psychological DV perpetration and victimization in young adulthood while controlling for childhood maltreatment; second, to determine whether the associations between IV and DV varied based on participants’ sex, socioeconomic status, and antisociality. To investigate this, data from a longitudinal, multi-informant, dual-cohort study in the Netherlands (TRracking Adolescents’ Individual Lives Survey) were used. Participants who self-reported their experiences of IV and whose romantic partners completed questionnaires on DV were included in the current sample (*N* = 522). Using hierarchical logistic regressions, results showed that IV exposure during childhood was not associated with DV perpetration or DV victimization during young adulthood. Further, this pattern of results did not vary as a function of sex, socioeconomic status, or antisociality. Overall, findings suggest that young adults in our sample demonstrate resilience against the intergenerational cycle of violence.

## Introduction

Dating violence (DV) is a widespread concern that encompasses physical, psychological, and sexual dimensions. While physical DV consists of the use of physical force (e.g., hitting) to hurt a dating partner, psychological DV refers to the use of verbal and nonverbal communication to exert control and/or emotional damage (Centers for Disease Control and Prevention, 2021). Recent data suggest that while 20% of adolescents experience physical DV (Wincentak et al., 2017), 40% report psychological DV in their lifetime (Ybarra et al., 2016). Further, contrary to popular opinion, significant overlap exists between DV perpetration and victimization. That is, many young adults who experience physical and psychological DV also inflict such forms of violence upon their partners (Kamimura et al., 2016). This overlapping pattern aligns with research indicating that certain risk factors may influence both DV perpetration and victimization (e.g., Paat & Markham, 2019). Relatedly, they suggest that adolescents may internalize and enact violent interactions as a whole, as opposed to mutually exclusive roles of victim and perpetrator (Park & Kim, 2019).

Counteracting DV is necessary to promote the well-being of young adults. Being a victim of physical and psychological DV is associated with adverse health outcomes (Eshelman & Levendosky, 2012; Exner-Cortens et al., 2013) and may negatively impact romantic partnerships in later adult life (Greenman & Matsuda, 2016). Therefore, identifying risk factors for DV perpetration and victimization is crucial to inform intervention development and promote the well-being of young adults. Exposure to interparental violence (IV) has been identified as a risk factor for DV; however, gaps in the literature exist. Additionally, little research has assessed moderators of the link between IV and DV. To overcome these gaps, this study assesses whether and among which individuals exposure to physical IV is associated with physical and psychological DV in young adulthood using a longitudinal design in a Dutch context.

### Interparental Violence and Dating Violence

The family environment is considered to influence the development of DV. According to Bandura’s (1977) social learning theory, individuals learn aggressive behavior through the observation of role models. In the context of IV, children may internalize interparental aggression as a legitimate way to resolve conflict in their future intimate relationships (Capaldi & Clark, 1998). Importantly, IV may not be just passively learned through exposure. Ehrensaft and Langhinrichsen-Rohling (2022) explain that witnessing IV may undermine adaptive psychosocial developmental outcomes (e.g., self-regulation, functional relational schemas), which consequently set youth in trajectories conducive to violence in future romantic relationships. In line with this, recent meta-analyses demonstrated that IV is consistently associated with both DV perpetration and victimization in young adulthood (Goncy, 2020), and that witnessing IV was among the strongest family-related predictors of DV perpetration and victimization (Hébert et al., 2019; Park & Kim, 2018).

Notwithstanding these results, several gaps in the literature remain. Many former studies are U.S.-based (Tomaszewska & Schuster, 2021), which hinders the generalizability of findings to other national contexts. Also, past research has relied on cross-sectional, single-informant, and retrospective designs (Goncy, 2020). Given that such methodological characteristics may inflate the strength of associations between IV and DV (Lindell & Whitney, 2001), research is needed to corroborate whether existing findings replicate among longitudinal, multi-informant studies. Additionally, former research has understudied the association between IV and psychological DV compared to that with physical DV, and failed to differentiate between distinct types of DV (Hébert et al., 2019). As such, research examining associations between IV and conceptually distinct types of DV (i.e., physical and psychological) is necessary. Finally, research evaluating the association between IV and DV while controlling for concurrent forms of family violence is scarce. Importantly, childhood maltreatment (i.e., violence from parental figures toward children) has also been identified as a risk factor for DV perpetration and victimization (Goncy et al., 2021). Childhood maltreatment is likely to co-occur with IV due to shared risk factors (Hamby et al., 2010; Jouriles et al., 2008). In fact, the associations between IV or childhood maltreatment and DV are mediated by shared underlying mechanisms involving emotional dysregulation (Iverson et al., 2014). That said, childhood maltreatment and IV represent qualitatively distinct forms of family violence; as such, examining both simultaneously is critical to obtaining a nuanced picture of DV family-based risk factors. Hence, research demonstrating the unique contribution of IV by controlling for co-occurring forms of family violence (e.g., childhood maltreatment) is needed.

### Interparental Violence, Dating Violence, and Variation Across Individuals

While IV exposure is positively associated with risk for DV, such associations may not be equal for all individuals. In other words, the intergenerational transmission of violence can vary across individuals and their social contexts. In particular, it can be hypothesized that men’s gender role socialization, socioeconomic adversity, and antisocial tendencies may enhance the association between IV and DV due to increased susceptibility to violent role modeling.

Researchers have highlighted the need to consider DV risk factors at different social-ecological levels beyond the individual (e.g., Dardis et al., 2015). Emerging ecological theoretical models of the intergenerational transmission of violence aim to capture interactions with intrapersonal, interpersonal, and social-structural risk factors to improve the explanatory power of existing models (Ehrensaft & Langhinrichsen-Rohling, 2022). However, little is known about risk factors that may enhance susceptibility to the intergenerational transmission of violence (Goncy, 2020). For these reasons, we intend to assess the moderating role of intrapersonal (e.g., antisociality) and social-structural vulnerabilities (i.e., gender and socioeconomic adversity) in our study. Since men are traditionally socialized to exert violence when resolving conflict, males may be more likely to internalize and model aggression than females (Malhi et al., 2020). Further, within families with a lower socioeconomic status (SES) background, exposure to stressors (e.g., financial stress) and a lack of resources (e.g., mental health support) may increase the susceptibility to violent role modeling (Foshee et al., 2008; O’Keefe, 1998). Finally, antisocial traits may undermine individuals’ abilities to self-regulate impulsive violent behavior (Estrada et al., 2021), which may also make them more susceptible to violent role modeling.

There is some empirical support for the moderating role of sex, SES, and antisociality in the association between IV and DV. Nevertheless, the literature is scarce, mixed, or focused on high-risk groups. Regarding sex and gender, the evidence is mixed and inconclusive. While recent meta-analyses reported no gender differences in the association between IV or childhood maltreatment and DV (Goncy, 2020; Goncy et al., 2021), earlier meta-analyses demonstrated a stronger association for men or women depending on the form of DV assessed (Smith-Marek et al., 2015; Stith et al., 2000). Concerning SES, literature is consistent yet scarce. For instance, among boys exposed to IV, those from lower SES families were more likely to report physical DV perpetration and victimization (O’Keefe, 1998). Similarly, the association between childhood maltreatment and physical DV perpetration was accentuated among adolescents with mothers from lower educational levels (Foshee et al., 2005). Finally, research showed that antisociality is a risk factor for partner violence victimization and perpetration among clinical samples (Spencer et al., 2019). Further, there is support for its moderation in community samples, although this research did not focus on IV exposure. For instance, individuals with antisocial traits showed greater susceptibility to social risk factors for DV perpetration, such as negative parent–child interactions (Goodnight et al., 2017).

### The Current Study

In response to the gaps in the literature outlined above, the current study uses a longitudinal, multi-informant approach to address two research aims: first, to assess whether exposure to physical IV before the age of 16 (retrospectively reported at age 19) predicts victimization and perpetration of both physical and psychological DV in young adulthood (6.6 years after reporting IV exposure) while controlling for childhood maltreatment; second, to determine whether sex, family SES, and antisociality moderate the association between physical IV and both physical and psychological DV. Based on theoretical notions and previous findings, several hypotheses were proposed. In relation to the main effects, it was expected that greater exposure to physical IV before the age of 16 would predict higher rates of both perpetration and victimization of physical and psychological DV in young adulthood. In regard to the moderation effects, male sex, lower family SES, and higher antisociality problems were hypothesized to enhance the association between physical IV and DV perpetration. However, given the significant overlap between reports of DV perpetration and victimization (Park & Kim, 2019), we hypothesized that these factors would also strengthen the link between IV and DV victimization to a lesser extent.

## Methods

### Participants

Data were derived from the *TRracking Adolescents’ Individual Lives Survey* (TRAILS) study (Huisman et al., 2008). TRAILS is an ongoing, longitudinal cohort study of Dutch adolescents followed from the age of 11 onwards. Individuals in two independent cohorts (i.e., community and clinical cohorts) completed six assessments over a 15-year period with a gap of 2 to 3 years between assessments. The clinical cohort includes individuals referred to a pediatric psychiatric outpatient clinic before the age of 11. Compared to the community cohort, the clinical cohort has a substantial overrepresentation of individuals with lifetime diagnoses of externalizing disorders at age 19, particularly attention-deficit/hyperactivity disorder and oppositional defiant disorder (Oldehinkel et al., 2015). For this study, a subsample of both cohorts was used which included adolescents whose romantic partners also participated when participants were 19 years old. Data from the first (T1; sex, family SES, and control variables), fourth (T4; IV, antisociality), and sixth assessments (T6; DV) were used in this study. T1 was conducted during 2001 and 2004 for the community and clinical cohorts, respectively. Only participants who answered questionnaires assessing experiences of IV at T4 (i.e., self-report) and whose romantic partners completed questionnaires on DV at T6 (i.e., partner-report) were included in the current sample. Participants from both cohorts who met inclusion criteria were combined to boost statistical power. A total of 522 participants were included in this study.

Overall, 58.6% of participants were female and 41.4% were male. In terms of ethnicity, the vast majority were Dutch (93.9%). Regarding sexuality, most participants self-identified as heterosexual (92.1%) followed by bisexual (5.6%) and homosexual (2.3%). Consistent with such trends, almost all participants for which partner data were available were in other-sex romantic relationships (97.8%) relative to same-sex relationships (2.2%). The mean age of participants across assessments were: T1 (*M* = 11.11 *SD* = 0.55), T4 (*M* = 19.03 *SD* = 0.62), and T6 (*M* = 25.66; *SD* = 0.64). For a detailed description of the sample, see Table 1. A comprehensive explanation of the TRAILS sampling procedure is described elsewhere (Huisman et al., 2008).

**Table 1.** Sample Descriptive Frequency Distributions Stratified by Cohort.

		Total Sample (*N* = 522)		Community Cohort (*n* = 441)		Clinical Cohort (*n* = 81)	
Variable	Level	*n*	%	*n*	%	*n*	%
Sex	Male	216	41.4	168	38.1	48	59.3
	Female	306	58.6	273	61.9	33	40.7
Ethnicity	Dutch	490	93.9	409	92.7	81	100.0
	Other	32	6.1	32	7.3	0	0.0
Family SES	Highest 25%	167	32.3	56	12.8	25	30.9
	Middle 50%	278	53.8	238	54.6	40	49.4
	Lowest 25%	72	13.9	142	32.6	16	19.8
Interparental Violence	Yes	69	13.2	59	13.4	10	12.3
	No	453	86.8	382	86.6	71	87.7
Child Maltreatment	Higher	76	14.6	67	15.2	9	11.1
	Lower	445	85.4	373	84.8	72	88.9
Antisociality	Above median	281	53.9	223	50.6	58	72.5
	Below median	240	46.1	218	49.4	22	27.5
Physical DVP	Yes	84	16.1	71	16.1	13	16.0
	No	438	83.9	370	83.9	68	84.0
Psychological DVP	Higher	87	16.7	76	17.2	11	13.6
	Lower	435	83.3	365	82.8	70	86.4
Physical DVV	Yes	82	15.7	71	16.1	11	13.6
	No	440	84.3	370	83.9	70	86.4
Psychological DVV	Higher	87	16.7	79	17.9	8	9.9
	Lower	435	83.3	362	82.1	73	90.1

*Note.* Frequencies may not add up to the total count due to missing values. SES = socioeconomic status; DVP = dating violence perpetration; DVV = dating violence victimization.

### Measures

#### Dating Violence

The Conflict in Adolescent Dating Relationships Inventory (CADRI; Wolfe et al., 2001) was used to assess both physical and psychological DV perpetration and victimization at T6. Specifically, the “physical abuse” (4-item) and “verbal emotional abuse” (9-item) subscales were analyzed separately for both perpetration and victimization assessments. Participants’ romantic partners reported their experiences of DV perpetration and victimization over the past year on a 4-point Likert-scale (*0* = *never* to *3* = *often [6 times or more]*). Participant-reported experiences of DV were not available. Regarding DV perpetration, sample items include: “*I threw something at him/her*” for physical abuse, and “*I did something to make him/her jealous*” for verbal-emotional abuse. Conversely, sample items for DV victimization include: “*He/she threw something at me*” for physical abuse, and “*He/she did something to make me jealous*” for verbal-emotional abuse. This measure showed good internal consistency for all subscales among the full cohort sample at T4; that is, physical abuse perpetration (Cronbach’s α = .70), physical abuse victimization (Cronbach’s α = .76), verbal emotional abuse perpetration (Cronbach’s α = .83), and verbal emotional abuse victimization (Cronbach’s α = .84). Due to the low prevalence in this study, physical abuse subscales were dichotomized as having experienced/perpetrated physical DV or not (*Yes* vs. *No*). However, the prevalence of verbal emotional abuse was considerably higher compared to physical abuse. Hence, verbal emotional abuse subscales were dichotomized using 16.7% of the highest scores as the cut-off point (*Lower* vs. *Higher*).

#### Interparental Violence

A measure of physical violence between parents developed by TRAILS researchers was used to assess physical IV exposure during childhood at T4. Participants were asked to retrospectively report experiences of witnessing IV before the age of 16 on a 4-item 5-point Likert-scale (*1* = *never* to *5* = *very often*). Sample items include: “*My mother/father has hit, kicked, bitten or punched the other person*” and “*My mother/father has used or tried to use a hard object, knife, or other weapon against the other person*.” This scale showed acceptable internal consistency among the full cohort sample at T4 (Cronbach’s α = .76). Considering the low prevalence of IV exposure in the current study, this scale was dichotomized as having witnessed IV or not (*Yes* vs. *No*).

#### Antisociality

The Adult Self-Report (ASR) from the Achenbach System of Empirically Based Assessment (ASEBA; Achenbach & Rescorla, 2001) was used to assess antisociality at T4. Specifically, the 20-item DSM-IV-oriented subscale on antisocial personality problems was selected. Participants were asked to report on the frequency and degree to which they conformed to antisocial traits and behaviors over the past 6 months on a 3-point Likert-scale (*0* = *not at all; 1* = *a little or sometimes; 2* = *clear or often*). Sample items include: “*I don’t feel guilty if I’ve done something I shouldn’t have done*” and “*I threaten people to hurt them*.” This measure showed good internal consistency among the full cohort at T4 (Cronbach’s α = .81). In this study, this scale showed a severely and positively skewed distribution. To deal with this, we used the median (*Mdn* = 0.1) as the cut-off point (*Below median* vs*. Above median*). The median value indicates that although our current sample included a clinical cohort, most adolescents reported virtually no symptoms of antisociality with a small minority reporting some to a lot.

#### Childhood Maltreatment

The parent-to-child aggression measure developed by TRAILS researchers assessed physical and emotional childhood maltreatment at T4. Participants were asked to retrospectively report experiences of maltreatment before the age of 16 on a 5-point Likert-scale (*1* = *never* to *5* = *very often*). Sample items include: “*My mother and/or father hit me with a belt, brush, stick or other hard object*” and, “*My mother and/or father said I would be sent away or that I had to leave the house*.” This measure demonstrated excellent internal consistency among the full cohort at T4 (α = .86). In the current sample, the scale was dichotomized due to a severely and positively skewed distribution indicating that most young adults reported low levels of child maltreatment. However, since the majority of participants reported some degree of maltreatment (i.e., scores above 1), scores were dichotomized into “Higher” (*n* = 76) or “Lower” (*n* = 445) as opposed to “Yes” (*n* = 396) or “No” (*n* = 125) to distinguish between minor and more severe histories of maltreatment. The differences in scores between childhood maltreatment and IV may be partly explained by the fact that our childhood maltreatment measure captures instances of violence that might be more prevalent compared to those assessed by our measure of IV (e.g., “*My mother and/or father called me stupid or lazy or something like that*” or “*My mother and/or father shook or pinched me*”).

#### Sex

A dichotomous variable was used to assess biological sex at T1 (*Male* or *Female*). This measure was used as a proxy for participants’ gender identity.

#### Family Socioeconomic Status

A composite indicator was used to measure family SES at T1. An average score encompassing standardized values for maternal and/or paternal educational level, professional occupation, and income was calculated. In this study, an ordinal variable was used by using the limits of the composite score’s interquartile range as cut-off points (*Lowest 25%, Middle 50%, Highest 25%*).

#### Sociodemographic Control Variables

Measures of age and ethnicity (*Dutch* vs. *Other*) at T1 were considered as control variables.

### Analytic Plan

For preliminary analyses, descriptive statistics (i.e., frequency distributions) were stratified by cohort (i.e., community vs. clinical), and chi-square tests were conducted to assess differences between participants across cohorts. A correlational matrix using Spearman’s *rho*, Cramer’s *V*, and point-biserial coefficients was created to evaluate unadjusted associations between all study variables.

The main analyses were conducted using stepwise complete case hierarchical logistic regressions separately for each of the four outcome variables (i.e., physical and psychological DV perpetration and victimization). In *Step 1*, only demographic variables associated with IV and/or DV outcomes were included. In *Step 2*, IV and childhood maltreatment were added. In *Step 3*, the interaction between IV and sex, family SES, and antisociality were included, as well as the main effect of antisociality (main effects of sex and family SES were already included in *Step 1*). We calculated McFadden’s pseudo *R*-squared for all models to illustrate their respective proportions of explained variance. Finally, sensitivity analyses were conducted using continuous instead of dichotomous variables, selecting a different antisociality cut-off, and removing the interaction terms to evaluate the robustness of the results. All analyses were performed in R 4.10 software using complete-case analyses.

## Results

### Preliminary Analyses

Differences between the community and clinical cohorts were assessed using chi-square tests of independence. The cohorts only differed in their distributions of participants’ sex (*χ*^2^[1, *N* = 522] = 11.78, *p* = .001) ethnicity (χ^2^[1, *N* = 522] = 5.06, *p* = .024), and antisociality (χ^2^[1, *N* = 522] = 12.24, *p* < .001). Participants who were male, reported Dutch ethnicity, and scored above the median on antisociality were overrepresented in the clinical cohort. For a stratified description of the sample, see Table 1.

Correlations between control, predictor, and outcome variables informed the decision to exclude control variables from the regression models (see Table 2). Neither age nor ethnicity was associated with predictors or outcomes. Hence, both sociodemographic indicators were excluded from the models. Physical IV was not associated with any form of DV; it was only weakly and positively correlated with antisociality, weakly and negatively correlated with family SES, as well as moderately and positively correlated with childhood maltreatment. Similarly, childhood maltreatment was not associated with any form of DV. Further, male sex was associated with higher partner-reported physical DV perpetration and lower partner-reported psychological DV victimization. All forms of DV were positively related to one another. In particular, reports of DV victimization and perpetration were strongly associated within each type of DV (i.e., physical and psychological).

**Table 2.** Spearman’s Rho, Cramer’s *V* and Point-Biserial Correlations With Confidence Intervals Between the Study Variables.

Variable	1	2	3	4	5	6	7	8	9	10
1. Interparental violence										
2. Physical DVP	−.00									
	[−0.09, 0.08]									
3. Psychological DVP	−.02	.35***								
	[−0.11, 0.06]	**[0.28, 0.43]**								
4. Physical DVV	.05	.57***	.34***							
	[−0.04, 0.13]	**[0.50, 0.62]**	**[0.26, 0.41]**							
5. Psychological DVV	.04	.37***	.54***	.41***						
	[−0.05, 0.12]	**[0.29, 0.44]**	**[0.48, 0.60]**	**[0.33, 0.48]**						
6. Child maltreatment	.31***	.04	.08	.02	.07					
	**[0.23, 0.38]**	[−0.05, 0.12]	[−0.01, 0.17]	[−0.07, 0.10]	[−0.02, 0.15]					
7. Antisociality	.15***	.08	.09*	.07	.11*	.19***				
	**[0.06, 0.23]**	[−0.01, 0.16]	**[0.01, 0.18]**	[−0.02, 0.16]	**[0.03, 0.20]**	**[0.10, 0.27]**				
8. Family SES	−.10*	−.07	−.05	−.03	−.02	−.07	−.04			
	**[−0.18, −0.01]**	[−0.15, 0.02]	[−0.14, 0.04]	[−0.12, 0.06]	[−0.11, 0.07]	[−0.16, 0.02]	[−0.13, 0.05]			
9. Sex	−.02	.12**	.01	−.04	−.08*	.01	.10*	.11*		
	[−0.06, 0.06]	**[0.03, 0.21]**	[−0.07, 0.04]	[−0.10, 0.03]	**[−**0**.16, −0.01]**	[−0.08, 0.03]	**[0.02, 0.19]**	**[0.02, 0.18]**		
10. Age	.00	.02	.00	−.00	−.04	.02	−.06	.04	.05	
	[−0.08, 0.09]	[−0.07, 0.10]	[−0.08, 0.09]	[−0.09, 0.08]	[−0.12, 0.05]	[−0.07, 0.10]	[−0.15, 0.03]	[−0.05, 0.12]	[−0.04, 0.14]	
11. Ethnicity	.02	.06	.01	−.02	.01	.10	−.00	−.06	−.02	−.01
	[−0.07, 0.06]	[−0.04, 0.15]	[−0.07, 0.05]	[−0.06, 0.04]	[−0.07, 0.05]	[−0.01, 0.21]	[−0.08, 0.03]	[−0.11, 0.04]	[−0.06, 0.05]	[−0.09, 0.08]

*Note.* DVP = dating violence perpetration; DVV = dating violence victimization; SES = socioeconomic status.

Bolded values indicate that confidence intervals exclude zero. *indicates *p* <.05. **indicates *p* <.01. ***indicates *p* <.001. Interparental violence (REF = No); Physical DVP (REF = No); Psychological DVP (REF = Low); Physical DVV (REF = No); Psychological DVV (REF = Low); Child maltreatment (REF = Low); Antisociality (REF = Below median); Family SES (Lowest 25% = REF; Middle 50% = 1; Highest 25% = 2); Sex (REF = Female); Age (years); Ethnicity (REF = Dutch).

### Regression Analyses

Stepwise logistic regression analyses were conducted to address the study objectives. First, analyses were performed to assess whether exposure to physical IV (T4) predicted physical and psychological DV perpetration and victimization 6.6 years after the reporting of IV exposure (T6). Second, analyses were conducted to determine whether sex, family SES, and antisociality moderated the association between IV and DV. Regression results from the fully adjusted models (*Step 3*) were chosen to be interpreted.

The first model aimed to predict partner-reported physical DV perpetration. Only sex emerged as a significant predictor in *Step 1* and *Step 2*. That is, compared to females, males’ partners were more likely to report physical DV perpetration (aOR = 1.86 [1.15, 3.03]). This implies that males more often are *victims* of physical DV from their partners than females. That said, this effect was not significant in *Step 3* and should be interpreted with caution. Importantly, the main effect of IV and the interaction effect between IV and antisociality were nonsignificant. For a full description of the model, see Table 3. The second model was conducted to predict partner-reported psychological DV perpetration. Only childhood maltreatment predicted this outcome in *Step 2*. Specifically, participants who had experienced higher levels of maltreatment were more likely to experience psychological DV from their partners compared to those who experienced lower levels (aOR = 1.93 [1.01, 3.57]). That said, the effect of childhood maltreatment dissipated in *Step 3*. For a full description of the model, see Table 4.

**Table 3.** Logistic Regression on the Association Between Physical Interparental Violence and Partner-Reported Physical Dating Violence Perpetration.

		Step 1		Step 2		Step 3	
Predictor	Level	aOR [95% CI]	*p*	aOR [95% CI]	*p*	aOR [95% CI]	*p*
Sex	Male (REF: Female)	**1.87 [1.15, 3.04]**	**.011**	**1.86 [1.15, 3.03]**	**.012**	1.63 [0.96, 2.78]	.070
Family SES	Highest 25% (REF: Lowest 25%)	0.64 [0.29, 1.44]	.268	0.64 [0.29, 1.44]	.269	0.75 [0.32, 1.87]	.525
	Middle 50% (REF: Lowest 25%)	1.18 [0.60, 2.46]	.646	1.17 [0.60, 2.44]	.661	1.26 [0.59, 2.97]	.568
Interparental Violence	Yes (REF: No)			0.91 [0.41, 1.86]	.805	2.17e−7 [4.16e−149, 5.41+05]	.986
Child Maltreatment	Higher (REF: Lower)			1.25 [0.61, 2.43]	.522	1.13 [0.53, 2.26]	.743
Antisociality	Above median (REF: Below median)					1.23 [0.73, 2.09]	.447
IV × Sex						2.31 [0.47, 1.40]	.324
IV × Family SES	Highest 25%					0.23 [0.01, 3.17]	.312
	Middle 50%					0.52 [0.07, 3.78]	.509
IV × Antisociality						6.78e+06 [3.51e−22, NA]	.985
		*R*^2^_McF_ = .05		*R*^2^_McF_ = .05		*R*^2^_McF_ = .08	

*Note.* The nonsignificant main effect of IV and the nonsignificant IV **×** Antisociality interaction effect yielded wide and uninterpretable 95% confidence intervals. SES = socioeconomic status; IV = interparental violence.

**Table 4.** Logistic Regression on the Association Between Physical Interparental Violence and Partner-Reported Psychological Dating Violence Perpetration.

		Step 1		Step 2		Step 3	
Predictor	Level	aOR [95% CI]	*p*	aOR [95% CI]	*p*	aOR [95% CI]	*p*
Sex	Male (REF: Female)	1.06 [0.66, 1.70]	.800	1.06 [0.65, 1.69]	.825	0.85 [0.50, 1.43]	.548
Family SES	Highest 25% (REF: Lowest 25%)	0.62 [0.31, 1.26]	.175	0.61 [0.30, 1.25]	.164	0.60 [0.28, 1.31]	.188
	Middle 50% (REF: Lowest 25%)	0.70 [0.37, 1.36]	.273	0.67 [0.36, 1.31]	.225	0.63 [0.31, 1.34]	.216
Interparental Violence	Yes (REF: No)			0.62 [0.27, 1.27]	.213	0.32 [0.01, 2.83]	.367
Child Maltreatment	Higher (REF: Lower)			**1.93 [1.01, 3.57]**	**.040**	1.72 [0.88, 3.24]	.102
Antisociality	Above median (REF: Below median)					1.57 [0.95, 2.64]	.085
IV × Sex						3.66 [0.71, 22.94]	.133
IV × Family SES	Highest 25%					0.62 [0.05, 6.24]	.690
	Middle 50%					0.58 [0.08, 4.25]	.581
IV × Antisociality						1.56 [0.22, 31.84]	.699
		*R*^2^_McF_ = .01		*R*^2^_McF_ = .02		*R*^2^_McF_ = .04	

*Note.* SES = socioeconomic status; IV = interparental violence.

The third model was performed to predict partner-reported physical DV victimization. No significant predictors were found for this outcome at any step of the model. For a full description of the model, see Table 5. Finally, the fourth model aimed to predict partner-reported psychological DV victimization. Only sex and antisociality predicted the outcome in *Step 3*. Specifically, males’ partners were less likely to report psychological DV victimization relative to females’ partners (aOR = 0.52 [0.29, 0.90]). This implies that males are less often *perpetrators* of psychological DV toward their partners than females. Likewise, compared to participants with lower antisociality problems, those with higher problems were more likely to perpetrate psychological DV (aOR = 1.72 [1.02, 2.94]). For a full description of the model, see Table 6. Overall, physical IV did not predict any form of DV across the four models. Furthermore, participants’ sex, family SES, and antisociality did not serve as moderators in the association between IV and any of the DV outcomes.

**Table 5.** Logistic Regression on the Association Between Physical Interparental Violence and Partner-Reported Physical Dating Violence Victimization.

		Model 1		Model 2		Model 3	
Predictor	Level	aOR [95% CI]	*p*	aOR [95% CI]	*p*	aOR [95% CI]	*p*
Sex	Male (REF: Female)	0.80 [0.48, 1.29]	.363	0.80 [0.48, 1.30]	.366	0.61 [0.35, 1.07]	.090
Family SES	Highest 25%	0.76 [0.37, 1.63]	.469	0.79 [0.38, 1.71]	.543	0.67 [0.30, 1.60]	.352
	Middle 50% (REF: Lowest 25%)	0.86 [0.44, 1.75]	.653	0.88 [0.46, 1.81]	.723	0.76 [0.36, 1.74]	.499
Interparental Violence	Yes (REF: No)			1.37 [0.68, 2.66]	.361	0.42 [0.04, 3.04]	.425
Child Maltreatment	Higher (REF: Lower)			1.01 [0.49, 1.97]	.972	0.86 [0.40, 1.73]	.690
Antisociality	Above median (REF: Below median)					1.40 [0.82, 2.40]	.215
IV × Sex						2.63 [0.64, 11.03]	.179
IV × Family SES	Highest 25%					2.10 [0.28, 16.65]	.468
	Middle 50%					1.16 [0.19, 7.74]	.869
IV × Antisociality						1.92 [0.38, 14.71]	.465
		*R*^2^_McF_ = .01		*R*^2^_McF_ = .01		*R*^2^_McF_ = .03	

*Note.* SES = socioeconomic status; IV = interparental violence.

**Table 6.** Logistic Regression on the Association Between Physical Interparental Violence and Partner-Reported Psychological Dating Violence Victimization.

		Model 1		Model 2		Model 3	
Predictor	Level	aOR [95% CI]	*p*	aOR [95% CI]	*p*	aOR [95% CI]	*p*
Sex	Male (REF: Female)	0.63 [0.38, 1.02]	.062	0.62 [0.38, 1.01]	.060	**0.52 [0.29, 0.90]**	**.022**
Family SES	Highest 25% (REF: Lowest 25%)	0.76 [0.38, 1.57]	.217	0.80 [0.40, 1.65]	.529	0.86 [0.39, 2.03]	.720
	Middle 50% (REF: Lowest 25%)	0.66 [0.35, 1.31]	.447	0.67 [0.35, 1.33]	.230	0.72 [0.34, 1.65]	.411
Interparental Violence	Yes (REF: No)			1.11 [0.55, 2.15]	.762	0.46 [0.02, 3.85]	.529
Child Maltreatment	Higher (REF: Lower)			1.56 [0.81, 2.91]	.168	1.31 [0.66, 2.50]	.423
Antisociality	Above median (REF: Below median)					**1.72 [1.02, 2.94]**	**.043**
IV × Sex						2.40 [0.55, 10.75]	.244
IV × Family SES	Highest 25%					0.62 [0.08, 4.44]	.639
	Middle 50%					0.52 [0.09, 2.89]	.454
IV × Antisociality						2.97 [0.45, 59.39]	.337
		*R*^2^_McF_ = .02		*R*^2^_McF_ = .03		*R*^2^_McF_ = .05	

*Note.* SES = socioeconomic status; IV = interparental violence.

### Sensitivity Analyses

Sensitivity analyses were conducted to evaluate the robustness of the results. First, logistic regressions were performed using continuous versions for IV, family SES, childhood maltreatment, and antisociality. In line with the previous results, these analyses yielded no significant effects for any of the fully adjusted models. Second, the initial logistic regression models were conducted with a different categorization for antisociality. Our chosen median-based categorization might be considered arbitrary and fail to differentiate participants with more extreme levels of antisociality. As such, the scale was dichotomized using 16.5% of the highest antisociality scores as the cut-off point. Using this approach, the patterns of association remained consistent; however, the significant main effect of antisociality on partner-reported psychological DV victimization dissipated. Finally, given the apparent redundancy of interaction effects, *Step 3* of the model was performed without interaction terms. Overall, the results were similar to those from the initial models with a few exceptions. In short, without testing for interactions, the main effect of sex on physical DV perpetration remained significant in *Step 3*. Regarding psychological DV perpetration, the main effect of childhood maltreatment remained significant and a marginally significant main effect of antisociality emerged in *Step 3*.

## Discussion

This study examined the longitudinal associations between IV and distinct types of DV perpetration and victimization (i.e., physical and psychological) in a Dutch context. The aim was to assess whether IV exposure before the age of 16 predicted DV in young adulthood, and the extent to which these associations were moderated by participants’ sex, family SES, and antisociality. The results showed that IV was not associated with physical or psychological DV perpetration or victimization 6.6 years after IV exposure was reported. Further, the association between IV and DV did not depend upon participants’ sex, family SES, and antisociality. Consistent with previous research, a strong positive association between reports of DV victimization and perpetration was found. In addition, a moderate positive association between IV and childhood maltreatment emerged.

### Why May Interparental Violence Not Be Associated With Dating Violence?

In contrast to what was expected, IV exposure did not predict physical or psychological DV perpetration or victimization in the current sample. This finding is inconsistent with previous research (Park & Kim, 2018). However, while links between IV and DV have been consistently found in former research, such associations were of small magnitude (Goncy, 2020; Hébert et al., 2019). In addition, the current study design differs in several aspects from most earlier studies. First, we used a longitudinal research design, whereas earlier research primarily used cross-sectional methods. Several meta-analyses demonstrated that associations between IV and DV were stronger in studies using cross-sectional than longitudinal designs (Haselschwerdt et al., 2019; Hébert et al., 2019). Relatedly, our study was partly prospective (i.e., the outcome was assessed 3 years after participants reported on their childhood exposure to IV), while most previous studies were retrospective. Previous research indicated that retrospective studies may overestimate the strength of associations (Lindell & Whitney, 2001). Second, in contrast to other studies, we dichotomized most of the variables because of restricted variance, which may have induced a loss of effect sizes (MacCallum et al., 2002). Third, using different informants for IV (i.e., self-report) and DV (i.e., partner-report) may entail different perceptions of interpersonal violence across informants. Consequently, such variations may have reduced the consistency of responses and thus account for the lack of associations. Nevertheless, given the multi-informant approach of the current study, it may have also minimized the influence of single-informant bias (Podsakoff et al., 2003). Overall, the current study can be construed as a relatively stringent assessment of the research question; therefore, it may have suppressed the emergence of weak, partly overestimated associations.

Taking into account these methodological considerations, the lack of associations in the current study suggests that only witnessing IV may not suffice to predict DV at later developmental stages. In other words, the intergenerational transmission of violence may only occur in combination with additional emotional or contextual vulnerabilities, such as psychological distress (Cascardi, 2016) or joint exposure to community violence (Margolin et al., 2009). While moderators in the current study did not influence the association between IV exposure and DV, examinations of alternative psychological and contextual moderators may shed light on intrapersonal and social-structural conditions accounting for the intergenerational cycle of violence.

Importantly, childhood maltreatment was associated with partner-reported psychological DV perpetration in the current sample. This association suggests that being the target of violence during childhood predicts the experience of psychological DV from a partner in young adulthood. This proposition aligns with previous research demonstrating that children’s victimization or injury during IV, compared to sole IV exposure, was linked to greater psychosocial maladjustment (Bayarri Fernàndez et al., 2011). Similarly, in contrast to IV exposure, childhood maltreatment was predictive of youth behavior problems (Margolin et al., 2009). Nevertheless, the association between maltreatment and DV dissipated in the final model following the addition of interaction terms.

### Why the Association Between Interparental and Dating Violence May Not Vary Across Sex, Family SES, and Antisociality

Overall, no subgroup differences in the link between IV and DV emerged in the current study. First, the relations between IV and DV were nonsignificant for both sexes. This suggests that neither males nor females may be susceptible to violent role modeling following IV exposure. Past research has documented mixed findings regarding the moderation of sex or gender in the link between IV and DV (Park & Kim, 2018; Smith-Marek et al., 2015). Relatedly, previous research has demonstrated that intimate partner violence in adult heterosexual relationships often entails reciprocal violence between male and female partners (Whitaker et al., 2007). From a social learning perspective, sex-specific propensities for violent role modeling may not have emerged in this study since both maternal and paternal figures can simultaneously perpetrate and be victims of violence. As such, there may be no strong empirical or theoretical grounds to hypothesize for or against sex-specific vulnerabilities in the intergenerational transmission of partner violence.

Importantly, sex differences in DV were found in this study. First, male sex decreased the likelihood of partner-reported psychological DV victimization. Given that over 97% of participants were in other-sex relationships, this result indicates that females were less likely to be victims of psychological DV than males. This finding contradicts previous research showing that females were at higher risk of psychological DV victimization than males (Exner-Cortens et al., 2021; Ybarra et al., 2016). Further, while the association between male sex and partner-reported physical DV perpetration was nonsignificant in the fully adjusted model, we found a trend showing that males’ dating partners were more likely to report physical DV perpetration than females’ partners. Again, considering that almost all participants were in relationships with the opposite sex, this finding aligns with cross-cultural research demonstrating higher rates of physical DV perpetration among female young adults relative to their male counterparts (Luo, 2021). Together, both patterns of association do not support the notion that men’s gender socialization (e.g., male superiority beliefs) may promote DV perpetration (Malhi et al., 2020). Instead, given that male-to-female DV may be generally perceived as more severe than female-to-male DV (Dardis et al., 2017), females may be more likely to enact or report instances of DV perpetration.

Second, inconsistent with the hypotheses, the relations between IV and DV were nonsignificant irrespective of participants’ SES. This finding contrasts with previous research reporting a higher risk for DV among low-SES youth exposed to IV (O’Keefe, 1998). While SES was negatively associated with IV in the current sample, it was not associated with childhood maltreatment or DV. Possibly, social policies in the Netherlands may grant youth from low-SES families access to resources (e.g., psychosocial services) needed to counteract the intergenerational transmission of violence. Alternatively, the time of measurement for family SES in this study (T1) may explain the lack of interaction effects. That is, young adults’ SES may matter more than their family’s SES when it comes to predicting DV.

Third, in contrast to expectations, the lack of associations between IV and DV did not vary based on participants’ antisociality. However, antisociality was positively associated with IV and childhood maltreatment in the current study. Previous research showed that children with antisocial traits showed greater vulnerability toward negative parent–child interactions (Goodnight et al., 2017). While these findings suggest that antisociality may increase susceptibility to violent role modeling in the context of adverse family environments, this effect may emerge when antisociality problems are clinically significant. In contrast to normative antisocial behavior in adolescence, clinical antisociality may trigger family violence in mutually reinforcing ways and consequently increase vulnerability (Moffit, 1993). Hence, the lack of moderation effects in this study may be explained by the fact that most participants did not meet subclinical or clinical criteria for antisociality. While further research is needed to evaluate this possibility, our sensitivity analyses did not show evidence in this direction. Alternatively, given the sharp decreases in antisocial behavior from adolescence to young adulthood (Moffitt, 1993), it is possible that the adverse effect of antisociality measured at T4 dissipated by the time DV was assessed at T6.

Nevertheless, compared to participants with lower antisociality problems, those with higher problems were more likely to perpetrate psychological DV against their partner. Put differently, the partners of participants with higher antisociality problems were more likely to report being victims of psychological DV. Similarly, previous research demonstrated that antisociality constitutes a risk factor for physical and psychological partner violence perpetration (Spencer et al., 2019). Possibly, higher rates of DV perpetration among individuals with higher antisocial tendencies may be explained by difficulties in self-regulating violent behavior or biases interpreting normative interpersonal conflict as threatening (Estrada et al., 2021).

### Limitations and Future Directions

This study had several methodological limitations. First, the sample’s demographic homogeneity hindered the generalizability of findings to ethnic minorities, non-heterosexual relationships, and gender non-conforming individuals. Further, even after combining the community and clinical cohorts, the vast majority of participants in our sample reported no exposure to IV and extremely low antisocial tendencies, thereby limiting generalizability to groups with higher clinical severity. Second, this study assessed physical IV and its associations with both physical and psychological DV. As such, associations between psychological IV and DV were not explored despite reported relations between psychological IV and maladjustment outcomes in previous research (Naughton et al., 2020). Relatedly, the gender of the perpetrator of IV was not assessed, thereby limiting our examination of gender-specific social learning pathways. Third, the single measurement of sex at T1 conflated conceptual distinctions between biological sex and self-reported gender identity. Fourth, assessments of family violence, antisociality, and DV were dichotomized due to the non-normality of distributions (Tripepi et al., 2008). Consequently, such dichotomizations may have obscured nuanced experiences of violence and symptom-related severity and frequency (Haselschwerdt et al., 2019). Finally, the unavailability of self-reported DV hindered our ability to evaluate the degree of consistency between partner- and self-reports. However, considering that few studies have integrated self- and partner-reported data, the multi-informant approach of the current study stands as a key methodological strength.

Based on such limitations, various scientific recommendations can be proposed. Future research should address the experiences of vulnerable groups at higher risk for DV, such as gender non-binary youth (Exner-Cortens et al., 2021). Further, assessing the effects of violence exposure beyond a single context (e.g., family and community) and distinguishing between gendered types of IV (e.g., father-to-mother vs. mother-to-father) may help provide more nuance to associated social learning processes (Margolin et al., 2009; Moretti et al., 2006). Relatedly, testing for the contributions of alternative moderators at multiple social-ecological levels is warranted. Regarding the research design, studies should assess concurrent forms of family violence (e.g., IV, maltreatment) while differentiating between distinct types of family violence and DV (i.e., physical versus psychological) to evaluate their respective implications for adjustment. At the same time, replicating our findings using alternative measures of IV and samples with a higher proportion of clinically referred at-risk youth is needed. Finally, in addition to risk factors, research should investigate the role of protective factors to inform strengths-based prevention efforts (Espelage et al., 2020).

### Theoretical and Practical Implications

Our results run counter to overly deterministic explanations of intergenerational partner violence transmission. The findings seem to indicate that young adults exposed to IV demonstrate to some extent resilience against the intergenerational cycle of violence. As such, young people may be capable of unlearning violence following exposure to IV. Additionally, our findings might indicate that social learning is prone to extinction over time; in other words, it may require more sustained exposure to the learned behavior across development.

Consequently, practitioners may need to avoid generalizing statements regarding violence in dating relationships among IV-exposed youth. While more research is needed to confirm these findings, the results indicate that counteracting learned behavior patterns stemming from IV exposure might be less effective than assumed in prevention efforts targeting DV in young adults. Further, key considerations should be incorporated during intervention development. That is, targeting youth with higher antisocial tendencies and implementing gender-sensitive approaches may maximize the impact of DV prevention efforts. Finally, practitioners must consider concurrent experiences of DV perpetration and victimization and thus address DV through a reciprocal approach.

## Conclusion

This study assessed the longitudinal associations between IV and DV, as well as the moderation of sex, family SES, and antisociality. The current findings did not support any association between IV and DV. Further, the lack of associations between IV and DV did not vary as a function of sex, family SES, or antisociality. Future research should aim to (a) replicate our results among community samples and vulnerable groups; (b) include measures of both physical and psychological IV; and (c) focus on the role of protective factors. While more research is warranted to confirm these findings, they suggest that young adults demonstrate resilience against the intergenerational cycle of violence.
